# Effect of Ninjin’yoeito on the Loss of Skeletal Muscle Function in Cancer-Bearing Mice

**DOI:** 10.3389/fphar.2018.01400

**Published:** 2018-11-30

**Authors:** Masahiro Ohsawa, Junya Maruoka, Chihiro Inami, Anna Iwaki, Tomoyasu Murakami, Kei-ichiro Ishikura

**Affiliations:** Department of Neuropharmacology, Graduate School of Pharmaceutical Sciences, Nagoya City University, Nagoya, Japan

**Keywords:** Ninjin’yoeito, cancer cachexia, insulin resistance, skeletal muscle, AMP kinase

## Abstract

Ninjin’yoeito (NYT), a traditional Japanese Kampo medicine formula, is used as a remedy for conditions, and physical weakness. Cancer cachexia is seen in advanced cancer patients and is defined by an ongoing loss of skeletal-muscle mass that leads to progressive functional impairment. In the present study, we examined the hypothesis whether NYT improves the functional loss of skeletal muscle cancer cachexia. Male C57/BL 6J mice with B16BF6 melanoma tumor showed decreased expression of myosin heavy chain (MHC) in the gastrocnemius muscle. Moreover, the expression of SOCS3 and phosphorylated STAT3 and AMPK was increased, and the expression of phosphorylated 4E-BP1 was decreased in the gastrocnemius muscle of tumor-bearing mice. These data suggested that amino acid metabolism was altered in tumor-bearing mice, which were normalized by the NYT intervention. The present study showed that NYT might be a novel therapeutic option for the treatment of sarcopenia occurring cancer cachexia.

## Introduction

Ninjin’yoeito (NYT) is a Japanese Kampo medicine used to improve recovery from diseases and other medical disorders, including fatigue, anemia, anorexia, night sweats, cold limbs, slight fever, chills, persistent cough, malaise, mental disequilibrium, and insomnia. Twelve crude herbs compose NYT: *Angelica acutiloba* roots (Japanese angelica root), *Atractylodes japonica* rhizomes (atractylodes rhizome), *Rehmannia glutinosa* var. *purpurea* roots (rehmannia root), *Wolfiporia cocos* sclerotia (poria sclerotium), *Panax ginseng* roots (ginseng), the bark of the trunk of *Cinnamomum cassia* (cinnamon bark), the pericarp of the ripe fruits of *Citrus unshiu* (citrus unshiu peel), *Polygala tenuifolia* root bark (polygala root), *Paeonia lactiflora* roots (peony root), *Astragalus membranaceus* roots (astragalus root), the fruit of *Schisandra chinensis* (schisandra fruit), and the roots and stolons of *Glycyrrhiza uralensis* (glycyrrhiza). NYT has been used traditionally to improve *qi* and blood deficiencies. NYT corrected total body weakness owing to anemia, slowed down the heart rate, and relaxed the body (Bensky et al., 2004).

Frailty is a common clinical syndrome in older adults that imparts an increased risk to poor health outcomes, including motor and mental disability (Fried et al., 2001). Frailty is triggered by several factors and the main reason is thought to be the dysfunction of skeletal muscle, called sarcopenia (Xue, 2011). Sarcopenia is the state of skeletal muscle tissue loss that is well observed in several disease such as obesity and diabetes (Cleasby et al., 2016). Sarcopenia attenuates the skeletal muscle function leading to the decline of physical activity (Evans, 2010). Malnutrition, caused by reduced activity and appetite, accelerates the progression of sarcopenia. The weakness originating from sarcopenia is a serious problem in the countries, which aim at healthy longevity in a super-aged society.

To evaluate the occurrence of sarcopenia in rodents, aged and disease models have been used. Particularly, the rodent cancer model showed extreme weight loss resulting from the skeletal muscle loss. In addition, this cancer model also showed complex disease symptoms during aging, called as cancer cachexia (Brennan, 1977). In cancer cachexia, food intake is reduced, and the reductions of protein and energy storage are evident due to the metabolic disturbance. Unlike other metabolic disturbances and aging animal models, our cancer cachexia model showed early reduction of skeletal muscle mass (Asp et al., 2010).

This evidence let us speculate that NYT could improve the symptoms of frailty. Therefore, we examined the effect of NYT on melanoma-induced cancer cachexia in mice.

## Materials and Methods

All animal experiments were approved by the Animal Care Committee of the Graduate School of Pharmaceutical Sciences, Nagoya City University, and were carried out in accordance to the guidelines of the National Institutes of Health and the Japanese Pharmacological Society.

### Animals

Male C57BL/6J mice (12 to 16-week-old; Japan SLC, Shizuoka, Japan) were used for each experiment. Mice were housed in a room with 5–6 mice in each cage, maintained at 23 ± 2°C with an alternating 12 h-light-dark cycle. Animals had free access to food and water, and were used only once in all the experiments.

### Cell Culture

B16F10 melanoma cells (American Type Culture Collection) were cultured in MEM enriched with 10% fetal bovine serum (FBS). When the cells were confluent, they were treated with trypsin/EDTA (0.05/0.02%) and detached. The trypsin/EDTA solution was recovered, neutralized with DMEM supplemented with 10% FBS and centrifuged at 500 ×*g* for 5 min. On day 0, 2.0 × 10^6^ cells suspended in 200 μL MEM were injected into the right flank, just under the skin of each mouse of the tumor group. An equal volume of MEM was injected into control group.

### Western Blotting

Quadriceps muscles were homogenized in RIPA buffer containing 50 mM Tris–HCl (pH 7.4), 500 mM NaCl, 5 mM NaF, 2 mM NaVo3, 1% NP-40, and 1 mM phenylmethylsulfonyl fluoride plus 250 μg/mL leupeptin, and 250 μg/mL aprotinin. The homogenate was then centrifuged at 13,000 ×*g* for 20 min at 4°C, and the resulted supernatant was retained as the sample. The protein concentration was measured using a Bradford assay kit (Bio-Rad Laboratories). Samples with equal amounts of protein (50 μg) were separated by SDS-PAGE (4–20%) and transferred to a nitrocellulose membrane (GE Health Life Science). The membrane was first blocked with 5% bovine serum albumin (Sigma-Aldrich, St. Louis, MO, United States) in Tris-buffered saline (pH 7.4), containing 0.05% Tween-20 (TBS/T), and then incubated with the primary antibody. The blots were visualized with enhanced chemiluminescence (SuperSignal West Dura Extended Duration Substrate; Thermo Fisher Scientific Inc., Suwannee, GA, United States) using LAS-3000 system (GE Healthcare Asia Co., Tokyo, Japan). The intensity of the band was analyzed and semi-quantified by computer-assisted densitometry using Image J. Each value was normalized by the respective value for GAPDH as an internal control.

### Drugs and Antibodies

Ninjin’yoeito was gifted from Kracie Pharma, Ltd. (Tokyo, Japan). NYT was prepared as a spray-dried powder of hot-water extracts from 12 species of crude drugs: Rehmannia root (4.0 g), Japanese angelica root (4.0 g), atractylodes rhizome (4.0 g), poria sclerotium (4.0 g), ginseng (3.0 g), cinnamon bark (2.5 g), polygala root (2.0 g), peony root (2.0 g), citrus Unshiu peel (2.0 g), astragalus root (1.5 g), glycyrrhiza (1.0 g), and schisandra fruit (1.0 g). The 3D-HPLC profile of NYT has already been reported (Ohsawa et al., 2011). Chemical markers, such as paeoniflorin, hesperidin, and glycyrrhizic acid, were used for quality control of NYT. NYT was freshly prepared by mixing in distilled water. Treatment with NYT oral supplementation at 1.0 g/kg was started from the day of cancer implantation. NYT was resolved in distilled water and all treatments were continued once a day for 14 days after cancer implantation. The antibodies used in this study were anti-Akt (Cell Signaling Technology), anti-Phospho Akt Ser479 (Cell Signaling Technology), anti-GSK-3β (Cell Signaling Technology), anti-Phospho GSK-3β Ser9 (Cell Signaling Technology), anti-STAT3 (Cell Signaling Technology), anti-Phospho STAT3 Tyr705 (Cell Signaling Technology), anti-SOCS3 (Abcam), anti-myosin heavy chain (R&B systems), anti-phospho AMP kinase (Cell Signaling Technology), anti-AMP kinase (Cell Signaling Technologies), anti-phospho 4E-BP1 (Cell Signaling Technology), anti-4E-BP1 (Cell Signaling Technology), anti-GAPDH (Cell Signaling Technology), anti-rabbit IgG HRP-linked (Cell Signaling Technology), and anti-mouse IgG HRP-linked (Cell Signaling Technology).

### Statistical Analysis

Data are expressed as mean with SEM. Student’s *t*-test was used to examine the differences between the two groups. Statistical significance of differences between multiple groups was assessed using *post hoc* Welch tests. Differences in probability values of less than 0.05 (*P* < 0.05) were considered statistically significant.

## Results

### Changes in the Body Components of B16BL6 Melanoma Cell-Transplanted Mice

**FIGURE 1:**
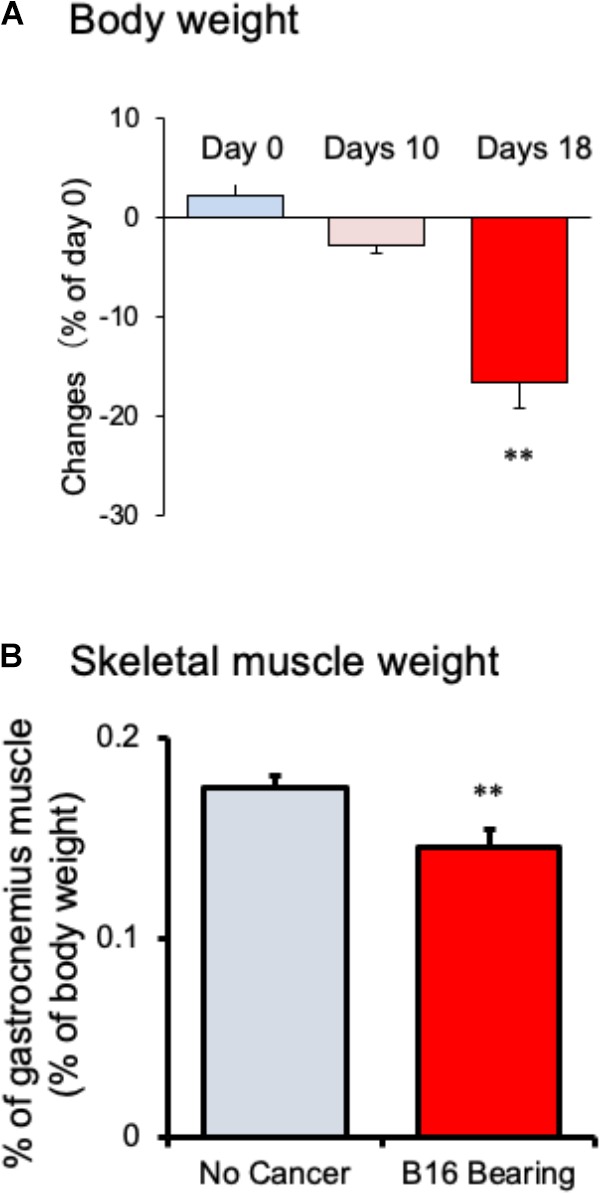
Changes in **(A)** body weight and **(B)** gastrocnemius skeletal muscle weight in mice with cancer cachexia. Samples were collected after 18 days of B16BF6 melanoma implantation. Each column represents the mean with SEM from 10 mice. ^∗∗^*P* < 0.01 vs. Day 0 or no cancer group.

Body weight of tumor-bearing mice gradually decreased from day 10 and showed a significant reduction after 18 days of melanoma implantation compared to that before melanoma implantation (Figure 1A; *n* = 10). The weight of the gastrocnemius muscle from tumor-bearing mice was decreased compared to that in no-cancer mice (Figure 1B; *n* = 10). Epididymal adipose tissue disappeared in tumor-bearing mice after 18 days of cancer implantation (data not shown). These changes in the tumor-bearing mice indicated that this mouse model effectively induced cancer cachexia.

### Changes in the Skeletal Muscle Protein Expression of Tumor-Bearing Mice

**FIGURE 2:**
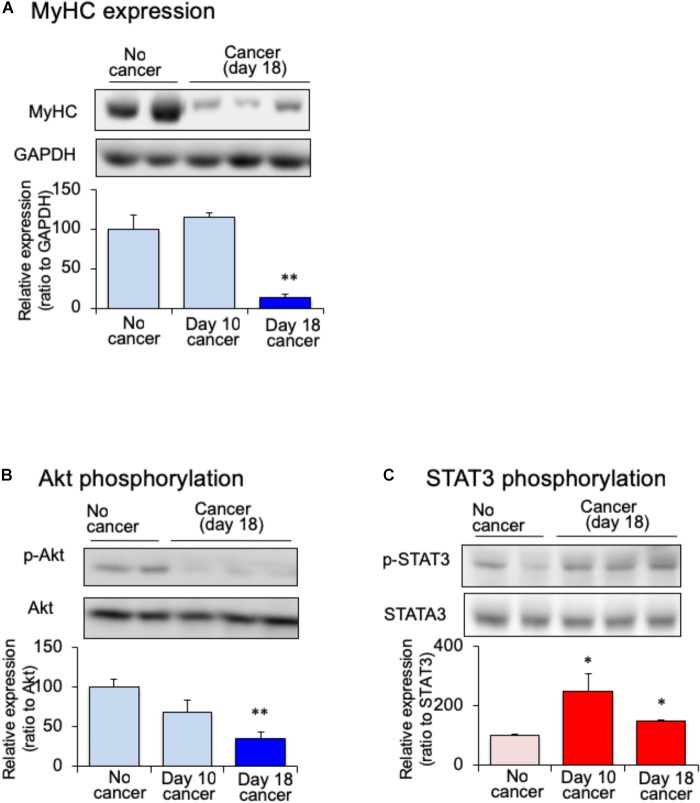
Changes of skeletal myosin heavy chain (MHC), **(A)** skeletal phosphorylated Akt **(B)** and skeletal phosphorylated STAT3 **(C)** in mice with cancer cachexia. Samples were collected after 10 or 18 days of B16BF6 melanoma injection. Each column represents the mean with SEM from six independent experiments. ^∗^*P* < 0.05 vs. no cancer group. ^∗∗^*P* < 0.01 vs. Day 0 or no cancer group.

Myofibrillar protein is lost at a faster rate than that of other proteins during skeletal muscle atrophy (Tisdale, 2009). Myosin heavy chain (MHC) is selectively depredated in cachectic phenotype (Fearon et al., 2012). To confirm the skeletal muscle atrophy in tumor-bearing mice, we examined the expression of MHC in the gastrocnemius muscle. The expression of MHC was lower in tumor-bearing mouse than in no-cancer mice after 18 days of melanoma implantation (Figure 2A). After 10 days of implantation, the expression of MHC did not vary between tumor-bearing and no-cancer mice. The reduction in the MHC expression was correlated with the reduction in gastrocnemius muscle weight of tumor-bearing mice. The expression of several signaling molecular regulates the maintenance of skeletal muscle proteins including insulin. Because cancer patients exhibit insulin resistance, the expression of Akt protein, a key component of insulin signaling, was examined. The expression of phosphorylated Akt in the gastrocnemius muscle was decreased in the tumor-bearing mice compared to that in no-cancer mice (Figure 2B). The decrease in phosphorylated Akt expression started 10 days after tumor implantation, and reached statistically significance at 18 days after tumor implantation. Then, we examined STAT3 expression, which interferes with insulin signaling. The expression of phosphorylated STAT3 in the gastrocnemius muscle was increased in tumor-bearing mice at 10 days after tumor implantation (Figure 2C). This increased expression of phosphorylated STAT3 was maintained even after 18 days of tumor implantation. The activation of STAT3 signaling preceded the attenuation of phosphorylated Akt expression.

### Effect of NYT on Body Changes in Tumor-Bearing Mice

**FIGURE 3:**
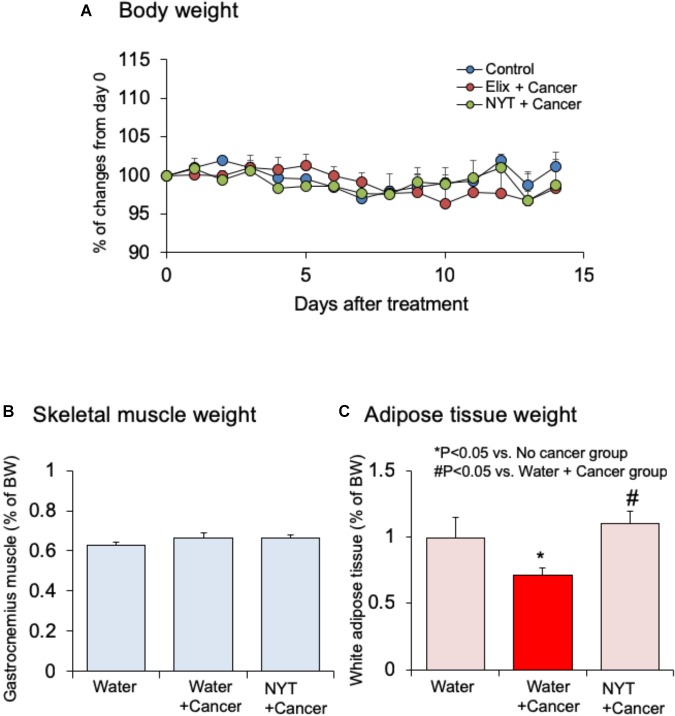
Effects of Ninjin’yoeito (NYT) on the alterations in **(A)** body weight, **(B)** gastrocnemius skeletal muscle weight, and **(C)** epididymal adipose tissue weight in mice with cancer cachexia. NYT was administered once a daily from 1 h before B16BF6 melanoma implantation and continued for 14 days. Each column represents the mean with SEM from 10 mice. ^∗^*P* < 0.05 vs. water-treated no-cancer group. ^#^*P* < 0.05 vs. water-treated cancer group.

Effect of NYT on the body weight, skeletal muscle mass, and adipose tissue mass in tumor-bearing mice were examined. Daily repeated oral treatment with NYT was started before 1 h of cancer implantation, and continued after 14 days of implantation. Body weight and gastrocnemius muscle weight did not change after 14 days of melanoma implantation (Figures 3A,B; *n* = 10). The weight of epididymal adipose tissue was reduced after 14 days of melanoma implantation (Figure 3C; *n* = 10). Daily repeated treatment with NYT (1 g/kg, p.o.) did not show significant influence on the body weight (Figure 3A), and skeletal muscle weight (Figure 3B) of tumor-bearing mouse. The epididymal adipose tissue weight in tumor-bearing mice was significantly increased after 14 days of repeated treatment with NYT (Figure 3C). The epididymal adipose tissue weight in NYT-treated tumor-bearing mice was comparable to that in no-cancer mice.

### Effect of NYT on the Expression of Intracellular Signaling Affecting Insulin Sensitivity in Skeletal Muscle

**FIGURE 4:**
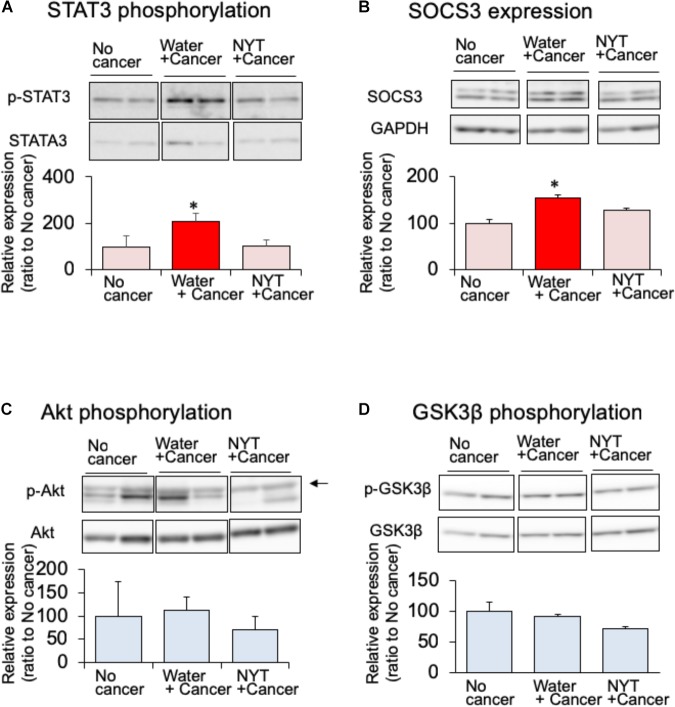
Effect of NYT on the phosphorylation of STAT3 **(A)**, SOCS3 expression **(B)**, phosphorylation of Akt **(C)**, and phosphorylation of GSK-3β **(D)** in the skeletal muscle of mouse with cancer cachexia. NYT was administered once a daily from 1 h before B16BF6 melanoma implantation and continued for 14 days. Samples were collected 24 h after last treatments. Each immunostaining obtained from same membrane. Each column represents the mean with SEM from three independent experiments. ^∗^*P* < 0.05 vs. water-treated no-cancer group.

Daily repeated treatment with NYT (1 g/kg, p.o.) did not show increased phosphorylation of STAT3 in the gastrocnemius muscle of tumor-bearing mice (Figure 4A). STAT3 induces the expression of its own feedback inhibitor, suppressor of cytokine signaling-3 (SOCS3). In the tumor-bearing mice, the expression of SOCS3 was increased after 14 days of melanoma implantation (Figure 4B). This increased SOCS3 expression was not observed by the daily repeated treatment with NYT (Figure 4B). These results suggested that NYT improved insulin signaling through the normalization of STAT3 signaling. Therefore, we examined the influence of daily repeated treatment with NYT on the insulin signaling molecular pathway in the skeletal muscle of tumor-bearing mice. Daily repeated treatment with NYT for 14 days did not affect the expression of phosphorylated Akt and GSK3β in the gastrocnemius muscle of tumor-bearing mice (Figures 4C,D).

### Effect of NYT on the Expression of Protein Synthesis Signaling Pathway in the Gastrocnemius Muscle of Tumor-Bearing Mice

**FIGURE 5:**
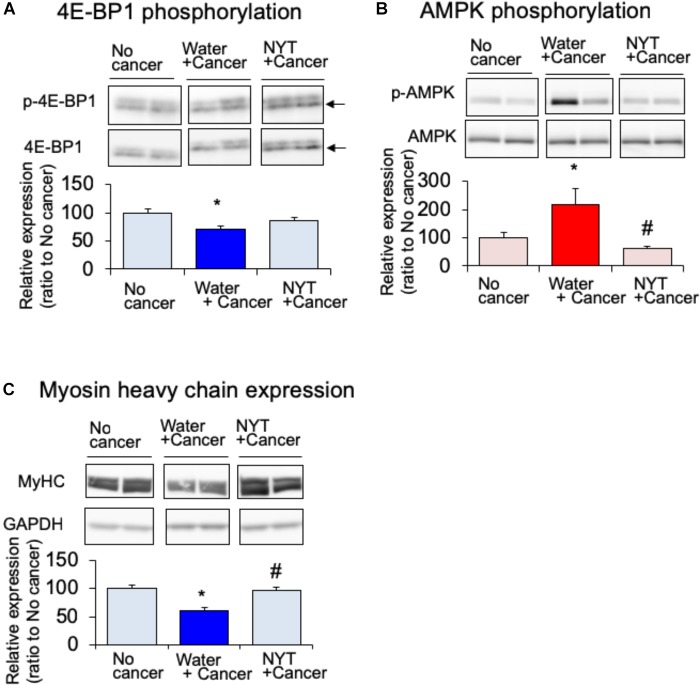
Effect of NYT on the phosphorylation of 4E-BP1 **(A)**, phosphorylation of AMP kinase **(B)**, and the expression of myosin heavy chain (MHC) **(C)** in the skeletal muscle of mouse with cancer cachexia. NYT was administered once a daily from 1 h before B16BF6 melanoma implantation and continued for 14 days. Samples were collected 24 h after last treatments. Each immunostaining obtained from same membrane. Each column represents the mean with SEM from six independent experiments. ^∗^*P* < 0.05 vs. water-treated no-cancer group. ^#^*P* < 0.05 vs. water-treated cancer group.

Amino acid metabolism is a key factor required for the maintenance of skeletal muscle protein. It is well known that mammalian target of rapamycin (mTOR) and eukaryotic translation initiation factor 4E-binding protein 1 (4E-BP1) regulate amino acid synthesis in the skeletal muscle (White et al., 2013). The activity of 4E-BP1 is regulated by mTOR-induced phosphorylation. The expression of phosphorylated 4E-BP1 after 14 days of melanoma implantation in the gastrocnemius muscle was significantly attenuated in tumor-bearing mice compared to that in the no-cancer group (Figure 5A). Such attenuated phosphorylation of 4E-BP1 was not evident in the daily repeated treatment with NYT (1 g/kg, p.o.) mice. mTOR signal is suppressed by 5′-adenosine monophosphate-activated protein kinase (AMPK) that is activated due to starvation of skeletal muscle (Bolster et al., 2002). The expression of the active form of AMPK (phosphorylated AMPK) was increased in the gastrocnemius muscle of tumor-bearing mice after 14 days of melanoma implantation (Figure 5B. Such increased phosphorylation was significantly attenuated by the repeated treatments with NYT. The reduction in MHC expression in the gastrocnemius muscle of tumor-bearing mice was ameliorated by the daily repeated treatment with NYT (Figure 5C).

## Discussion

The present study indicated that NYT maintained the skeletal muscle mass through the improvement of dysfunction of amino acid metabolism in cancer cachexia. Such NYT-induced improvement of amino acid metabolism dysfunction might be because of the inhibition of activated STAT3 and AMPK signaling.

Skeletal muscle is considered as the important amino acid storage tissue, and almost 50% of amino acid is stored in this tissue. Skeletal muscle mass is regulated constantly by the balance between catabolism and anabolism. The muscle protein synthesis is suppressed under cachexia in rodents (White et al., 2011). The present study indicated that the attenuation of phosphorylation of 4E-BP1 is responsible for the loss of protein synthesis in skeletal muscle. Moreover, the expression of MHC was greatly decreased in the skeletal muscle of tumor-bearing mice. Based on these findings, the attenuation of protein synthesis might be involved in the muscle atrophy of tumor-bearing mice.

Mammalian target of rapamycin signaling is regulated by several intracellular signaling molecules. It is well known that Akt signaling, which is activated by insulin, stimulates mTOR signaling. On the other hand, STAT3, which is activated by inflammatory cytokines, inhibits mTOR signaling (Bonetto et al., 2011). The present study showed that STAT3 signaling is activated in the skeletal muscle of tumor-bearing mice, and this activation is evident before the onset of skeletal muscle loss. STAT3 is strongly phosphorylated by the activation of GP130, which is receptor of interleukin-6 (IL-6), IL-11, leukemia inducing factor, and oncostatin M (Gearing et al., 1992). IL-6 is released under inflammation and is involved in the insulin resistance of obesity or diabetes (Klover et al., 2003). In obesity and diabetes mellitus, adipocyte enlargement and subsequent cell death leads to the infiltration of immune cells into adipose tissue (Ogawa and Kasuga, 2008). Infiltrated immune cells into adipose tissue can release inflammatory cytokines. Because the released inflammatory cytokines affect the function of adipocytes, IL-6 is also released from adipocytes. It is possible that STAT3 signaling might be activated by the enhanced release of IL-6 that is mediated by the functional alterations of adipocytes. Therefore, the attenuation of mTOR signaling in the skeletal muscle of tumor-bearing mice might be caused by the activation of STAT3 signaling through the enhanced production of adipocyte IL-6.

IL-6 also regulates the activity of AMPK. Overexpression of skeletal IL-6 induces AMPK phosphorylation, in addition to the phosphorylation of STAT3 (White et al., 2013). AMPK is a serine/threonine kinase, which is activated by the increase in intracellular AMP/ATP ratio, and regulates the master-switch of glucose and lipid metabolism. It is also reported that AMPK regulates protein synthesis and stimulates the degradation of myofibril proteins (Nakashima and Yakabe, 2007). AMPK-induced protein degradation has been suggested to be mediated by the inhibition of mTOR signaling or promotion of protein ubiquitination (Bolster et al., 2002). The present study also indicated that AMPK is activated in the skeletal muscle of tumor-bearing mice. Therefore, it is hypothesized that the activation of AMPK is partly involved in the reduction of skeletal muscles of tumor-bearing mice.

Daily repeated treatments with NYT normalized STAT3 signaling in the skeletal muscle of tumor-bearing mice. Moreover, NYT treatment also normalized the reduction of phosphorylated 4E-BP1 expression and enhanced the phosphorylation of AMPK in the skeletal muscle of tumor-bearing mice. On the other hand, NYT treatment did not affect Akt signaling, which is involved in the protein synthesis of skeletal muscle. Based on these results, because STAT3 activates AMPK signaling (White et al., 2011), we hypothesized that maintenance of skeletal muscle protein by NYT might be because of the normalization of the alterations of the intracellular signal transduction suppressing the maintenance of skeletal muscle mass originated from the activation of STAT3 signaling.

The dosage of NYT has been fixed from human clinical doses. The therapeutic dosage of NYT for human prescription authorized by Ministry of Health, Labor and Welfare of Japan is 6.7 g/day. The adequate amount of oral NYT dose for mice was determined to be 1.0 g/kg (Miyamoto et al., 2017).

In the present study, the mechanisms of STAT3 activation in the skeletal muscle have not been clarified. The adipose tissue weight of tumor-bearing mice was less than that in no-cancer mice. This reduced adipose tissue weight was reversed by the daily treatment with NYT. It is possible that NTY might protect the adipose tissue from the influence of cancer. Because the activated immune cells and enlarged adipocytes release inflammatory cytokines that activates STAT3 signaling, NYT-induced inhibition of STAT3 phosphorylation might be the reduction of inflammation by protection from adipose tissue reduction. Indeed, it has been reported that increased inflammatory cytokines, such as IL-6, might be involved in the enhanced phosphorylation of STAT3 in muscle tissue of cancer cachexia model (Bonetto et al., 2011). It is reported that NYT ameliorates chronic inflammation in chronic kidney disease (Hsiao et al., 2015). These reports support our hypothesis that NYT inhibits the inflammation in the adipose tissue of tumor-bearing mice.

**FIGURE 6:**
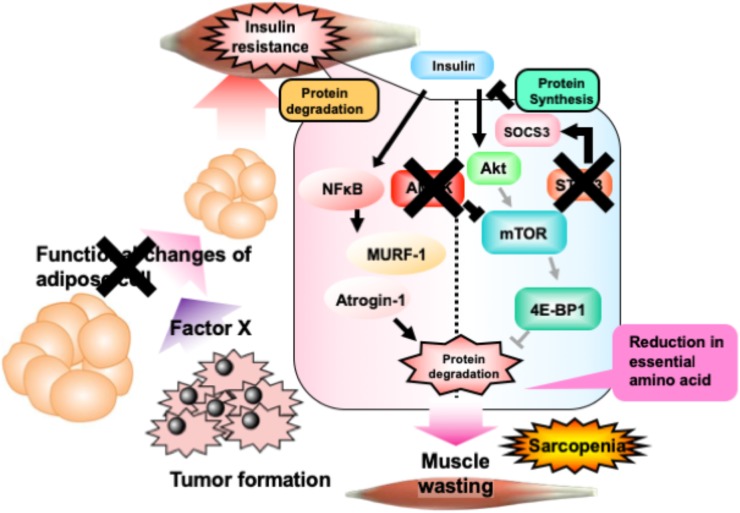
Schematic representation of the effect of NYT on the melanoma-induced cancer cachexia in mice. Details are provided in the text. In brief, phosphorylation of AMP kinase in cancer cachexia mouse skeletal muscle is caused by the increase of inflammatory cytokines, tumor-necrosis factor-α, free fatty acids which is released from adipocytes. Moreover, these changes might be decreased the phosphorylation of 4E-BP1 in skeletal muscle of mice with cancer cachexia. NYT normalizes the increased phosphorylation of AMP kinase and the decreased phosphorylation of 4E-BP1 through the functional changes of adipocyte and inhibition of STAT3 signaling. The action site of NYT indicated as cross mark. Bold lines indicate enhancement in cancer cachexia model. Pale colored lines indicate the reduced signaling in cancer cachexia.

The present study indicated the ameliorative effect of NYT on cancer-induced sarcopenia. As summarized in Figure 6, cancer cells produce an unknown factor that induces reduction of adipose tissue. Such alterations in adipose tissue induce skeletal muscle insulin resistance through the stimulation of STAT3 signaling, followed by the activation of AMPK. Activation of AMPK reduces overall contents of essential amino acids in the skeletal muscle through the inhibition of mTOR-4E-BP1 signaling. Daily treatment with NYT protects the adipocytes, followed by the reduction of STAT3 and AMPK signaling. This NYT-induced protection might be involved in the amelioration of muscle atrophy in cancer cachexia model. This effect of NYT might be because of the improvement of alterations of anabolism and catabolism in the skeletal muscle cells. NYT might improve the capacity of amino acid storage in the skeletal muscle.

## Author Contributions

K-iI, CI, AI, and JM performed the experiments and statistical analysis. TM performed the statistical analysis and revision of manuscript. MO designed this study, wrote the manuscript, and performed the statistical analysis.

## Conflict of Interest Statement

Kracie Pharma, Ltd. (Tokyo, Japan) provided research funds and Ninjin’yoeito, which is a product of Kracie Pharma, Ltd. The authors declare that the research was conducted in the absence of any commercial or financial relationships that could be construed as a potential conflict of interest.
